# The ratio of ADSCs to HSC-progenitors in adipose tissue derived SVF may provide the key to predict the outcome of stem-cell therapy

**DOI:** 10.1186/s40169-018-0183-8

**Published:** 2018-02-07

**Authors:** Mehmet Okyay Kilinc, Antonio Santidrian, Ivelina Minev, Robert Toth, Dobrin Draganov, Duong Nguyen, Elliot Lander, Mark Berman, Boris Minev, Aladar A. Szalay

**Affiliations:** 1 Department of Biochemistry, Biocenter, University of Wuerzburg, Am Hubland, 97070 Würzburg, Germany; 2 StemImmune Inc., San Diego, CA 92122 USA; 3 Radiation Medicine, Moores Cancer Center, University of California San Diego, La Jolla, CA 92037 USA; 4 Cell Surgical Network and California Stem Cell Treatment Center, Rancho Mirage, CA 92270 USA; 5 Virbio, Inc., Parlin, NJ 08859 USA

**Keywords:** Adipose, Stromal vascular fraction (SVF), Adipose-derived stromal/stem cells (ADSCs), Expanded mesenchymal stem cells, Cell therapy, Stem cell treatment

## Abstract

**Background:**

Stromal vascular fraction (SVF) represents an attractive source of adult stem cells and progenitors, holding great promise for numerous cell therapy approaches. In 2017, it was reported that 1524 patients received autologous SVF following the enzymatic digestion of liposuction fat. The treatment was safe and effective and patients showed significant clinical improvement. In a collaborative study, we analyzed SVF obtained from 58 patients having degenerative, inflammatory, autoimmune diseases, and advanced stage cancer.

**Results:**

Flow analysis showed that freshly isolated SVF was very heterogeneous and harbored four major subsets specific to adipose tissue; CD34^high^ CD45^−^ CD31^−^ CD146^−^ adipose-derived stromal/stem cells (ADSCs), CD34^low^ CD45^+^ CD206^+^CD31^−^ CD146^−^ hematopoietic stem cell-progenitors (HSC-progenitors), CD34^high^ CD45^−^ CD31^+^CD146^+^ adipose tissue-endothelial cells and CD45^−^CD34^−^CD31^−^CD146^+^ pericytes. Culturing and expanding of SVF revealed a homogenous population lacking hematopoietic lineage markers CD45 and CD34, but were positive for CD90, CD73, CD105, and CD44. Flow cytometry sorting of viable individual subpopulations revealed that ADSCs had the capacity to grow in adherent culture. The identity of the expanded cells as mesenchymal stem cells (MSCs) was further confirmed based on their differentiation into adipogenic and osteogenic lineages. To identify the potential factors, which may determine the beneficial outcome of treatment, we followed 44 patients post-SVF treatment. The gender, age, clinical condition, certain SVF-dose and route of injection, did not play a role on the clinical outcome. Interestingly, SVF yield seemed to be affected by patient’s characteristic to various extents. Furthermore, the therapy with adipose-derived and expanded-mesenchymal stem cells (ADE-MSCs) on a limited number of patients, did not suggest increased efficacies compared to SVF treatment. Therefore, we tested the hypothesis that a certain combination, rather than individual subset of cells may play a role in determining the treatment efficacy and found that the combination of ADSCs to HSC-progenitor cells can be correlated with overall treatment efficacy.

**Conclusions:**

We found that a 2:1 ratio of ADSCs to HSC-progenitors seems to be the key for a successful cell therapy. These findings open the way to future rational design of new treatment regimens for individuals by adjusting the cell ratio before the treatment.

## Background

The use of adipose tissue as a source of MSCs has become advantageous for cell-based therapy approaches, due to their easy accessibility, higher cell yields, and in vitro proliferative and multilineage differentiation capacity [1, 2]. Adipose-derived stem cells have regenerative potential and exhibit anti-inflammatory, immunomodulatory, and pro-angiogenic effects [3–5]. Because of these distinctive characteristics, SVF, which includes ADSC, holds a great promise in regenerative medicine and tissue engineering [6, 7]. Therapeutic applications of these cells in patients suffering from orthopedic conditions such as bone and cartilage defects, osteoarthritis, soft and hard-tissue defects, cardiovascular disorders, skin and wound defects, and auto-immune disorders have been documented with significant beneficial use and improvements as reported in some of the clinical trials [8–13]. Drs. Berman and Lander have recently published their safety and clinical assessment data gathered from a large number of patients (close to 1500) with various medical conditions using both IV and regional deployments of SVF [14]. Their data showed both safety and a good clinical outcome using a closed sterile surgical lipotransfer procedure. SVF can be freshly isolated from stroma lying within adipose tissue and blood vessels, and clinically used as autologous cells without further in vitro manipulation on the same day that the adipose tissue was collected.

In contrast to the hematopoietic stem cell’s (HSC) biology, where the hierarchy of differentiation is well established, the complex nature of stromal stem/progenitor cells biology remains a wide-open venue for discovery. Therefore, new researches focus on the characterization of the stem/progenitor and/or immature MSC-like cell properties and the identification of the microenvironmental factors, which regulate them. SVF is very heterogeneous and contain ADSCs and hematopoietic precursors, mature vascular endothelial and progenitors cells, pericytes, fibroblasts, granulocytes, monocyte/macrophages, and lymphocytes [15]. Characterization of SVF revealed the majority of the cells being either positive for CD45 (also known as a leukocyte common antigen) or CD34 which is a well-known stem cell marker in both hematopoietic and endothelial lineages. For more specific cell characterization, a combination of markers such as CD31 (endothelial marker) and CD146 (perivascular marker) is necessary to assess cell identity and their frequency [16, 17]. These studies also revealed that CD34^+^ cells displaying characteristics similar to MSC dominate the stem/progenitor components. These ADSCs surround the outer ring of the vasculature by forming a supra-adventitial layer, which are colonized on their surfaces by CD146^+^ pericytes [18, 19]. The CD34^+^CD31^+^ ECs fraction is associated with the luminal layer and was shown to exhibit the ability to form functional blood vessels in vivo. It has been shown that adipose tissue-ECs have a different gene expression profile as well as limited in vitro expansion potential in comparison to blood derived endothelial progenitor cells (EPCs) [20, 21]. Adipose-derived and expanded-mesenchymal stem cells (ADE-MSCs) can be isolated from SVF by in vitro cultivation on plastic surfaces, which exhibit a spindle-like morphology similar to fibroblast [22]. Although ADE-MSCs acquire a homogenous phenotype CD90^+^, CD73^+^, CD105^+^, CD45^−^, CD34^−^ during in vitro culture, initial expression levels in the freshly isolated SVF are low [22, 23]. They have self-renewal potency and ability to give rise to at least adipogenic, osteogenic, and chondrogenic lineages. Furthermore, there is evidence that these cells can generate a variety of other cell types including cardiomyocytes [24]. Despite some controversy, studies suggest that classical CD34^+^ ADSCs, also pericytes and HSC-progenitors can give rise to ADE-MSCs and convey multipotency [25, 26]. The cause of contradiction is believed to be due to the differences in isolation methods as well as post-culture conditions [27].

Although there is still no clear consensus on the benefit of culture expanded ADE-MSCs over freshly isolated SVFs, additional in vitro manipulation steps used to create extensive numbers of ADE-MSCs in autologous setting are costly, require regulatory approval, and could lead to contamination and early-differentiation problems diminishing therapeutic potency. As such, the success of SVF therapy in clinical settings clearly depends on the understanding of the mechanisms of action of ADSCs and other cellular components after infusion. Using SVF cells rather than ADSCs may bring more significant benefits and healing potential because of the inclusion of heterogeneous cell types and different factors with paracrine effects [28]. Recent review articles have displayed that the major mode of action for MSCs is the establishment of a regenerative microenvironment in response to tissue injury [29, 30]. When delivered by IV, SVF cells are able to recruit into areas of inflammation through the vascular system. They specifically target those areas. When the damaged tissue stimulate the stem cells, they start secreting an array of growth, anti-apoptotic, anti-inflammatory and angiogenic factors, and possibly differentiating into tissue cells [31].

Previous research has highlighted the difficulty of concluding the therapeutic potential for a given application by just looking at SVF’s heterogeneity, yield, isolation method, and patient’s characteristics. Recently, a detailed biomarker analysis has been proposed as a clinical predictor, which was found to be inconclusive because of common phenotype distribution among distinct subpopulations [32]. As abundantly addressed in the last couple of years, there is a great need to evaluate, identify, and optimize the potential factors to provide optimal clinical response in stem cell treatment [33]. Therefore, in this study, we particularly looked for the potential factors, which may determine the best therapeutic effect. We evaluated the composition and clinical efficacy of freshly isolated autologous SVF cells from donors with various medical conditions and tested them in further analysis by culture expansion. We followed 44 SVF-treated patients who received different SVF-dose with defined combination, and found that a certain combination of ADSCs to HSC-progenitor cells could be correlated with overall treatment efficacy. To our knowledge, this is the first report to reveal such correlation for the effectiveness of autologous treatment. We believe these novel findings will pave the way to more effective therapeutic cell preparations resulting in better clinical outcomes.

## Methods

### Study population and procedure

Patients applied to California stem cell treatment centers (Cell Surgical Network) and all participants signed an IRB-approved consent form. Liposuction aspirates were obtained under local anesthesia using an IRB-approved protocol (International Cell Surgical Society; IRB# ICSS-2016-024) from donors as previously explained in details [14]. In short, patients received local anesthesia consisting of lidocaine 0.5% with epinephrine. They underwent liposuction procedure utilizing 2.5–3 mm cannula.

### Patient evaluation

To assess subjective outcomes, standard questionnaires and evaluation system for follow-ups were utilized. Details about which questionnaires were used were published previously [14]. Data were collected via e-mail and telephone and entered into a customized TrackVia (Denver, Colorado) database. All responses were voluntary and patients did not receive compensation to participate. To assess the potential benefit of the treatment, a benefit index by grading the improvements on a scale 1–5 based on the patient reports was generated. Each of these numbers was assigned by the principal investigators based on review of the following subjective outcomes tests for each condition. For category#1 (orthopedic conditions), the following tests were administered to patients: Visual analog pain scale plus, knee injury and osteoarthritis outcome score, WOMAC Score, Hip injury and osteoarthritis outcome score, disability arm shoulder hand score, foot and ankle outcomes questionnaire, oswestry back questionnaire, neck disability index. For category #2 (organ and tissue dysfunction including neurologic disease, cardiac disease and urologic disease), the following tests were administered for each specific condition: AQoL-4, cardiac status form, Minnesota heart failure questionnaire, erectile hardness grading form EHGS, Peyronie’s bother score, pelvic urgency frequency form and O’Leary-Sant form. For Category 3 (Autoimmune function), the following tests were administered: autoimmune status form and AQoL-4.

### Adipose-tissue processing, isolation of SVF and injection

Stromal vascular fraction cells were isolated following established protocols [14]. 50 cubic centimeter (cc) of adipose tissue was collected into a TP-101 syringe (single use sterile fat processing syringe) and condensed by centrifugation at 2800 rpm for 3 min in the Time Machine centrifuge. Good manufacturing practices (GMP) grade Collagenase (Roche GMP grade, Heidelberg, Germany) containing 12.5 Wünsch units was added and the lipoaspirate was placed on a Time Machine shaker/incubator in 37 °C for 30 min to enzymatically digest the collagen matrix in order to procure the SVF inside closed Time Machine syringes (TP-102 syringe by MediKhan, Los Angeles, USA) in the operating room. Collagenase from Roche is assayed in Wünsch units. One (Collagen Degrading Units) CDU catalyzes the hydrolysis of 1 μmol of l-leucine equivalents from collagen in 5 h at 37 °C, pH 7.4 and 1 Wünsch unit = ~ 1000 CDU. The Time Machine™ (USA trade name for the MediKhan Lipokit system—Los Angeles, CA, USA; 510(k) approved for fat grafting) is a specialized closed surgical processing system for isolating stromal vascular fraction. It combines a centrifuge and incubator to prepare SVF in a closed system. The end product of 5–10 ml SVF was collected using a sterile 20 ml Luer Lock syringe (Sigma-Aldrich, St Louis, MO, USA). The patient’s SVF is then injected back into their body, either directly into the inflamed area (such as the knee or hip joint) or into the bloodstream, via an intravenous injection. SVF deployments in most orthopedic cases were I.V and intra-articular [14].

### Flow analysis

Immunophenotyping, cell count and cell viability of freshly isolated SVFs were performed by flow cytometry on a 4-color FACSCalibur flow cytometer (Becton–Dickinson). Removal of erythrocytes was carried out by using RBC lysis buffer (GIBCO). The following monoclonal antibodies (MAbs) conjugated to various fluorochromes were used in single cell suspensions of SVFs: CD45 (clone HI30), CD34 (clone 8G12), CD90 (clone 5E10), CD31 (clone WM59), CD73 (clone AD2), CD14 (clone M5E2), CD206 (clone 19.2), CD105 (clone SN6), CD44 (clone L178), CD66b (G10F5), CD235a (GA-R2 (HIR2)), CD3 (clone HIT3a), CD19 (clone HIB19), CD56 (clone B159). All antibodies were purchased from BD Biosciences, except the anti-human CD105 (clone SN6), which was purchased from e-biosciences, and staining was performed according to standard staining protocol as described before [34]. Cell viability was verified by 7-amino-actinomycin D (7-AAD) or Propidium Iodide (PI) and Annexin-V staining (BD Biosciences).

### Culture of SVF

A portion of freshly isolated SVF cells was plated in a CELLstart CTS (Gibco)-coated plate with culture media. The serum-free culture media was composed of DMEM (Invitrogen), 5% Stemulate (Cook Regentec, IN, USA), 1 × GlutaMAX (Invitrogen), MEM non-essential amino acids, and Pen/Strep (Invitrogen). Stemulate is a GMP grade pooled human platelet lysate, which supports the expansion of multiple types of cells including MSCs. It has essential growth factors and other proteins such as platelet-derived growth factors (PDGF-AA, PDGF-AB/BB), epidermal growth factor (EGF), vascular endothelial growth factor (VEGF), basic fibroblast growth factor (bFGF), transforming growth factor beta 1 (TGF-B1), fibrinogen, regulated upon activation, normal T -cell expressed, and secreted (RANTES), and chemokine ligand 5 (CCL5), C-X-C Motif Chemokine Ligand CXCL1/2/3 that are necessary for cell growth and functions. Primary cells were cultured for 7–14 days and were defined as “Passage 0” (P0). The medium was replaced every 3 days, and cells were passaged every week or two when 80% confluence was reached. 1 × TrypLE Express (Life Technologies) was used to dissociate cells. Immunophenotyping on different passages were carried out as described above.

### Mesodermal lineage differentiation assays

The ability of cells to differentiate into mesodermal lineages was tested in adipogenic or osteogenic differentiation mediums provided by iXcells Biotechnologies. Differentiation was initiated by seeding MSC at a density of 20,000 cells per cm^2^. After 1–2 weeks of culture in defined media conditions, cells were stained with Oil Red O (Sigma) for the detection of lipids and Osteoimage Mineralization Dye (Lonza) to visualize osteogenic mineralization. The quantification of intracellular lipid droplets was performed using AdipoRed Assay kit from Lonza, following the instructions provided by the manufacturer. Fluorescence measurement was carried out at Tecan Infinite 200 plate reader with excitation at 485 nm and emission at 572 nm. For the quantification of mineralization, Osteoimage Mineralization Assay was utilized (Lonza). Excitation and emission wavelengths were 492 and 520, respectively.

### Cell sorting

Cells were processed and incubated with the following antibodies, CD45, CD235a, CD34, CD31 and CD146, as described above. The viability dye PI was added before sorting for exclusion of dead and apoptotic cells. Cell sorting was performed using on a FACSAria III cell sorter (BD Biosciences) equipped with a class I biosafety cabinet. Four-way sorting was performed at 10,000-to-20,000 events per minute. Sorted populations were placed into flat bottom 48-well plates coated either with fibronectin (Millipore) or CELLstart CTS (Gibco). DMEM + 5% Stemulate culture media was used to culture sorted cells. Viability was determined by Trypan blue exclusion on day 5–7 post-culture.

### Data and statistical analysis

The data were analyzed by comparing a “benefit index” of the treatment to various patient factors such as age, gender, body mass index (BMI), dosage, and route of injection (further described in the proceeding sections). Table 1 contains the raw benefit index numbers for each patient. The ratios of various cell subtypes were also calculated, and compared to the various patient factors in order to determine which, if any, factors were correlated to treatment efficacy.

**Table 1:** List of 44 SVF-treated patient’s gender, age, medical reason, delivery methods and benefit index

Patient (P) number (#)	Gender: F/M	Age	Medical reason for SVF Rx/(classification category)	Delivery method: direct (localized) injection (DI) or I.V.	Benefit index
P#1	F	65	Knee & poliomyelitis (1)	IV + DI	4
P#2	M	81	Knee (1)	IV + DI	2
P#3	F	20	Bladder disease (2)	IV	5
P#4	M	70	Optic nerve ischemia (2)	IV	2
P#5	M	84	Cardiac dysfunction (2)	IV	N/A
P#6	F	40	Multiple sclerosis (MS) (2)	DI	3
P#7	M	50	Autoimmune disease (3)	IV	N/A
P#8	F	69	Hip (1)	IV + DI	4
P#9	F	50	Knee (1)	IV + DI	4
P#10	F	79	Arthritis (1)	IV + DI	4
P#11	F	43	Arthritis (1)	IV + DI	N/A
P#12	F	56	Arthritis; hip (1)	IV + DI	3
P#13	F	77	Arthritis; shoulder (1)	IV + DI	3
P#14	F	56	Knee (1)	IV + DI	3
P#15	M	64	Arthritis; knee& shoulder (1)	IV + DI	4
P#16	M	62	Peyronie’s (2)	DI	3
P#17	M	79	Degenerative joint disease; hips and knees (1)	IV + DI	4
P#18	M	57	Arthritis; knee (1)	IV + DI	4
P#19	F	46	Fibromyalgia (2)	IV + DI	N/A
P#20	F	47	Autoimmune; lupus (3)	IV + DI	2
P#21	M	62	Renal dysfunction (2)	IV	1
P#22	F	37	Optic neuritis (2)	IV	4
P#23	F	56	ALS (amyotrophic lateral sclerosis) (2)	IV	1
P#24	M	91	Stroke (2)	IV	3
P#25	M	55	Spinal cord injury &arthritis; shoulder (1)	IV	4
P#26	M	79	Arthritis; knee (1)	IV + DI	1
P#27	F	58	Arthritis; foot (1)	IV + DI	2
P#28	F	82	Wellness & memory (2)	IV	1
P#29	F	61	Arthritis; knee (1)	IV + DI	N/A
P#30	F	45	Arthritis; knee (1)	IV + DI	1
P#31	F	65	Arthritis; knee (1)	IV + DI	1
P#32	F	63	Gout (1)	IV	4
P#33	M	59	Knee (1)	IV + DI	4
P#34	F	76	Arthritis; hip (1)	IV + DI	1
P#35	M	66	Arthritis; hip (1)	IV + DI	3
P#36	F	66	Shoulder (1)	IV + DI	3
P#37	M	62	Shoulder & knee (1)	IV + DI	N/A
P#38	M	56	Renal dysfunction (2)	IV	N/A
P#39	F	16	Torn achilles tendon (1)	IV + DI	N/A
P#40	M	54	Lower motor neuron disease (2)	DI	1
P#41	M	62	Arthritis; hip& knee (1)	IV + DI	3
P#42	M	68	Knee (1)	IV + DI	3
P#43	M	67	Arthritis; knee & shoulder (1)	IV + DI	1
P#44	F	66	Lower back pain (1)	IV + DI	2

## Results

### Effect of gender, age, clinical condition and route of injection on the clinical outcome post-SVF treatment

Most of the human clinical studies regarding implantation of autologous adipose SVF containing ADSCs have specifically assessed effectiveness and safety, without identifying the factors that might be responsible for potential benefits. Therefore, in this study we analyzed patient’s characteristics and SVF prepared by using standard procedures in order to assess the post-treatment clinical outcome. The autologous SVF was returned back to the donor patients with various disease conditions such as orthopedic, inflammatory, degenerative tissue or organ, and autoimmune diseases within 2–3 h. All patients underwent treatment with SVF cells as scheduled and no complications related to adipose tissue processing and SVF cells preparation was noticed. There were no reported side effects associated with SVF cell therapy. We followed the patients every 3–4 months for about a year post-treatment, to assess the potential benefit of the treatment. All patients were requested to fill out a questionnaire, and we generated a benefit index by grading the improvements on a scale 1–5 based on the patient reports.

Grade numbering:

5-Complete recovery

4-A large difference (A noticeable & continuous difference)

3-Some improvements

2-Minimal improvement

1-No improvement.

**Fig. 1:**
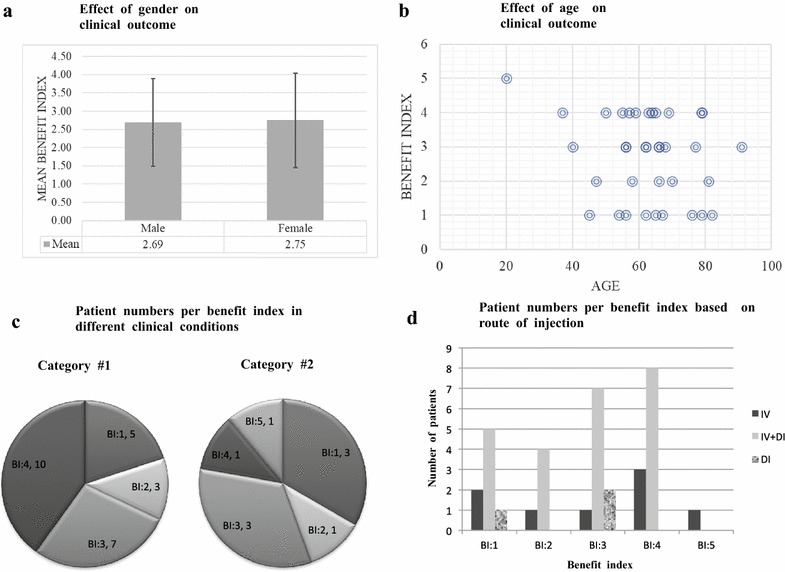
Correlation between clinical outcome and gender (**a**), age (**b**), clinical conditions (**c**), different routes of injections (**d**). **a** The mean benefit index is shown as the height of the bars for males and females, with the error bars representing the 95% confidence interval. **b** A scatter plot of the age versus benefit index is shown. **c** The breakdown of distribution of the benefit index in various clinical conditions, where Category #1 represents orthopedic applications and Category #2 represents organ or tissue dysfunctions. **d** The number of SVF-treated patients with various routes of injection is shown as the height of the bars, with the benefit index shown on the X-axis

Aiming to identify clinical factors predictive of response to SVF therapy, we first asked whether gender of the patients played a role in the effectiveness of treatment. Table 1 list all of the patient’s gender, age, medical reason, delivery methods and benefit index. Out of 24 females, 4 patients did not participate in the follow-up program (N/A), and 5 patients reported no benefits (25%). Furthermore, 1 patient with “Complete recovery” (5%); 6 patients with “A big difference” (30%); 5 patients with “Some improvement” (25%), and 3 patients with “Minimal improvements” (15%), were detected respectively. For male patients, we observed almost an identical trend; Among 16 male patients, 12 patients benefited at various degrees (Complete recovery: 0%, A big difference; 31%, Some improvement; 31%” and Minimal improvements; 13%), whereas 4 patients did not report any benefit (25%). A bar graph, in Fig. 1a shows the mean benefit index for gender with 95% confidence interval. The mean benefit index was 2.69 for male versus 2.75 for female. Therefore, we argue that the gender did not play a role in the patients reported therapy outcome. We subsequently analyzed whether the age of the patients was a contributing factor. The treatment age started with the youngest patient who was 16 years old, and the oldest patient who was 91 years old. The majority of patients were between 40 and 79 years old. The largest population of patients, at 15, was in the age group 60–69, followed by 11 patients in the age group of 50–59, 6 for 70–79, and 5 for 40–49. There was only 1 patient for each of the following groups: < 20; 20–29; 30–39 and 90–99. When the ages of all patients were plotted against the benefit index for direct correlation, we could not see any difference for the mean benefit index among the different ages (Fig. 1b). Therefore, we concluded that age could not be a specific determining factor for treatment success. Next, we divided the patients into three main classification categories based on their clinical conditions to investigate whether any particular disease or condition benefitted most from the treatment. All of the orthopedic applications along with joint associated chronic pain, stiffness, deformity, decrease in the range of motion, and arthritis were included in category #1 (Table 1). Organ or tissue dysfunctions were grouped under category #2, and autoimmune diseases were grouped under category #3. We further indicated the route of SVF injection for each patient in our analyses: IV injection alone (I.V.), direct localized injection alone (D.I.), or both. We had the highest number of patients in category #1 with 29, followed by 13 and 2 patients for category #2 and #3, respectively. We analyzed the number of patients within category #1 using the benefit index scale from 1 to 5, and compared the data with the distribution of category #2. Furthermore, we analyzed and compared the route of injections with their different benefit index. The data analyses indicated that neither the specific clinical condition nor the route of injection played a significant role in clinical outcome since patients from both of the categories, #1 and #2, and with different route of injections, could be found in every benefit index to almost the same extent. Taken together, the administration of the autologous SVF for the treatment of different medical conditions has a positive effect, but the factors such as gender, age, clinical condition, and route of injection did not play a decisive role in the treatment efficacy nor are the proof of the true underlying mechanism of improvement.

### Characterization of cell types in SVF

**Fig. 2:**
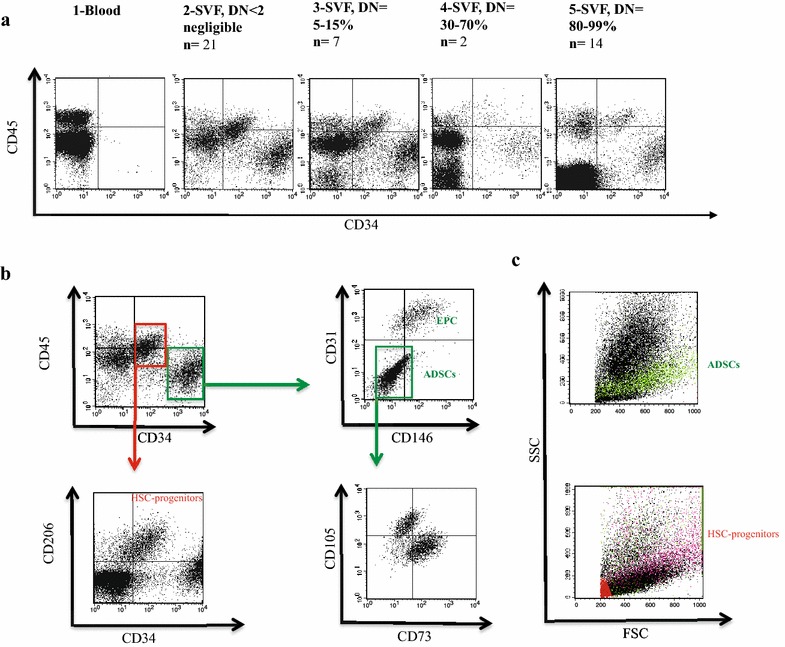
Flow cytometry analysis of SVF. **a** shows a representative of CD45 and CD34 dot blot analysis for the different DN ratios. A1 is the blood analysis shown for the comparison and n represents the number of patient’s samples with the given percentage. **b** Detailed analysis of subpopulation phenotype. Arrows point the gating and each identified phenotype was marked with the corresponding nomenclature. **c** Back-gating analysis of ADSCs and HSC-Progenitors on forward and side scatter dot plot to show their cellular profile

Next we investigated whether the stem/progenitor cells composition and dose of SVF could show a correlation with treatment efficacy. Initially, we hypothesized that the presence of one of the major cell subsets injected at optimal numbers would favor the observed improvements. Earlier studies reported the heterogeneity in SVF and great variability in the percentages of different subsets [35, 36]. Some reports have focused mostly on the difference in the isolation methods, handling, variability of cell yields and mode of administration [37, 38]. To date, there are insufficient data to establish a correlation between SVF-dose, and direct therapeutic effect. Therefore, we conducted a comprehensive analysis by characterizing the cells types with regenerative potential present in the donor SVF samples. To determine the composition of SVF, we performed flow cytometry analysis and identified 4 distinct cell populations based on the surface expression of CD45 and CD34 (Fig. 2a). Two of these populations which are CD34^−^ CD45^high^ and CD34^−^CD45^low^, were also present in the blood (Fig. 2a1). On the other hand, the CD34^+^ positive subsets: CD34^high^ CD45^−^ and CD34^low^ CD45^+^, were only detected in the SVF samples. In 21 out of 44 patients the combined ratio of all 4 populations was almost 100 percent of the nucleated cells. In these patients, the average percentage of CD34^−^ CD45^high^, CD34^−^CD45^low^, CD34^high^ CD45^−^, and CD34^low^ CD45^+^ was 16, 44, 28, 12%, respectively (data not shown). In 18 patients, on the other hand, the percentage of double negatives (DNs; CD34^−^ CD45^−^) showed distinct variations in three different ranges; 5–15% (n = 8), 30–70% (n = 2), and 80–99% (n = 13). Figure 2a panel 2 through 5 shows a representative analysis of CD34 and CD45 for each of these groups. The variation in the presence of fibroblasts, pre-adipocytes, smooth muscle, mature endothelial cells and the remaining RBCs following the lysis is believed to be the main contributor for the differences of DNs among patients.

We further investigated SVF samples, in more detail using cell type specific surface markers in patient samples. There is not a unique marker for CD34^+^ cell subsets, therefore a combination of markers were used for more comprehensive analyses. Figure 2b shows the phenotype of the subsets based on flow cytometry analysis. CD31 (PECAM-1) is a classic marker for endothelial cells and their progenitors. In combination with CD146, we distinguished CD34^high^ CD45^−^ CD31^+^CD146^+^ AT-ECs separately from CD34^high^ CD45^−^CD31^−^ CD146^−^ ADSCs within the CD34^high^ cell subset. The fraction of EC compartment among CD34^high^ subset displayed a great variability by ranging from 1 to 55% with an average of 21%, across all of the donors with DN < 2. On the other hand, CD34^low^ CD45^+^ cells, co-expressed CD206 and CD14. This subset has been described as Hematopoietic/Monocyte-like progenitor cells (HSC-progenitors) in the literature [39–41]. Back-gating analysis of this subset on forward and side scatter (FSC and SSC) dot plot revealed that they reside on top of ADSCs indicating they are more granular (Fig. 1c). Heterogeneous expression of CD105 and CD73 was detected among SVF samples. Figure 1b shows a representative of well-separated subsets within the ADSCs; CD34^+^CD105^+^ CD73^−^ and CD34^+^105^−^CD73^+^ cells. The percentage of pericytes described as CD45^−^CD34^−^CD31^−^CD146^+^ cells made up only a small fraction (average percentage of nearly 1%) of whole SVF. CD45^+^ cells were equally abundant in SVF, and the majority of these cells were CD66b^+^ Neutrophils (data not shown). Other observed immune cell populations included B-cells, NK cells, and T-cells and it is highly likely they were derived from blood vessels co-harvested during the procedure. The viability of CD34^+^ cells was verified regularly by 7-AAD/PI and Annexin-V staining and they were 80–90% viable post-isolation.

In conclusion, the following four main adipose-resident subsets were identified in SVF samples: ADSCs: CD34^high^ CD45^−^ CD31^−^ CD146^−^, HSC-progenitors: CD34^low^ CD45^+^ CD206^+^CD31^−^ CD146^−^, AT-ECs: CD34^high^ CD45^−^ CD31^+^CD146^+^, and Pericytes: CD45^−^CD34^−^CD31^−^CD146^+^. We believe that the abundance of one or some of these subsets in combination reflecting some level of orchestration were the primary factors which determine the treatment outcome.

### Patient characteristics and their effect on SVF yield

**Fig. 3:**
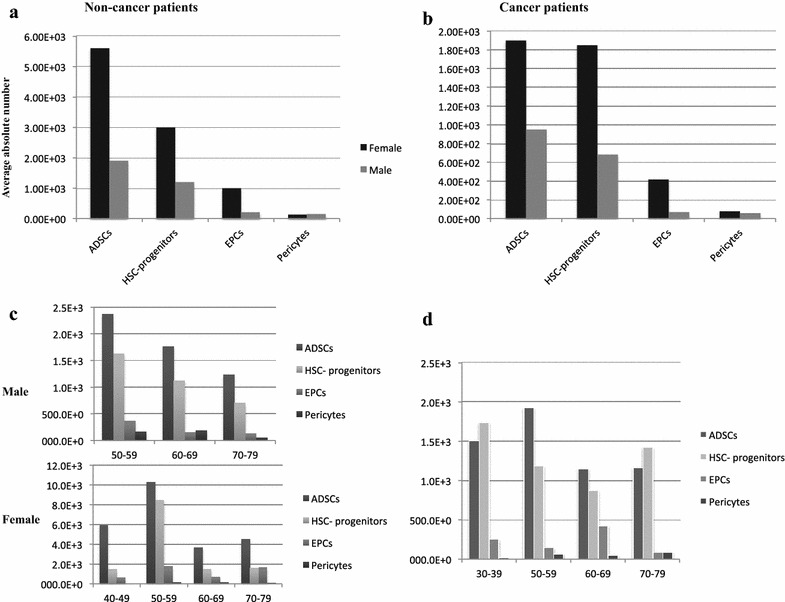
Evaluation of SVF yields based on gender, cancer and age. **a** Trends between the total numbers of 4 cell subsets freshly isolated from fat among female and male participants in (**a**) non-cancer patients versus (**b**) cancer patients can be seen by comparing the height of the bars. **c** The mean number of the subsets analyzed per age in males (upper figure) versus females (lower figure) in non-cancer patients is shown as the height of the bars. (Note that the age groups with less than one patient are not included in the analysis.) **d** The mean number of the subsets (the height of the bars) is compared for the various different age groups (the X-axis) for observing trends

Few previous studies compared some of the patient factors such as age, gender, BMI that might affect the yield, composition, purity and potency of the freshly isolated SVF samples, reported controversial results [35, 42–45]. To determine whether the patient’s disease conditions may alter the SVF’s yield and composition, we also included 14 cancer patients. Enumeration analyses lead us to evaluate the possible effect of tumor growth on the cell composition of SVF and further assess the feasibility of utilizing SVF in cancer patient’s treatment. Identical liposuction procedure was followed for such patients and samples were analyzed for the presence of the same 4 subsets of cells. Due to the variations in the ratio of double negatives among all of the patients (non-cancer and cancer), we calculated the absolute number of isolated cell subsets as shown in Table 2 and then performed a correlation analysis between patient characteristic and the SVF yield. We first checked the gender difference for a possible factor in harvested stem cells; ADSCs and HSC-progenitors. We found that the average absolute numbers of ADSCs in females (5.6E+3/per cc of fat/non-cancer) was almost threefold higher than the average yield in male patients (1.9E+3/per cc of fat/non-cancer) (Fig. 3a). A similar difference was observed with the absolute number of HSC-progenitors: 3E+3/cc of fat of HSC-progenitors in females versus 1.2E+3 cells, in males. Regarding the absolute number of AT-ECs, the difference between males and females was fivefold: 1E+3/cc ECs in females versus 212E+0 cells, in males. No difference was observed for pericytes. Interestingly, a similar trend was observed in between 8 male and 6 female cancer patients, although all of the 4 cell subsets were overall lower (at least two–threefold) in number compared to non-cancer patients (Fig. 3b). The average absolute number of ADSCs, HSC-progenitors, AT-ECs and pericytes in female versus male cancer patients were 1.9E+3 versus 948E+0, 1.9E+3 versus 682E+0, 417.5E+0 versus 70.3E+0, 75.3E+0 versus 56.3E+0, respectively. Furthermore, different age categories in 9-year increments were made. In females, the treatment age range was 16–82 years old and marked the end points of eight different age groups. There were no patients in the age group of 20–29 and a single patient in each of these groups: 10–19, 30–39 and 80–89. Therefore, the yield of 4 SVF subsets were calculated in four different groups, each with more than 3 patients. The average total number of ADSCs was 5.9E+3 in the age group between 40 and 49, reached its highest, 10.3E+3, in the age group 50–59, and declined in the subsequent groups: 3.7E+3 (60–69); 4.5E+3 (70–79). For HSC-progenitors, the highest average total number was recorded in the same group 8.5E+3 (50–59) but remained almost the same (1.5E+3 and 1.6E+3) in the other age groups. In AT-ECs and pericytes, the distribution of average count per age showed the following patterns: 658E+0/(40–49), 1.8E+3/(50–59), 700E+0/(60–69), 1.7E+3/(70–79), and 82E+0/(40–49), 165E+0/(50–59), 158E+0/(60–69), 93E+0/(70–79), respectively. On the other hand, in males treatment ages started very late, around the age of 50–59 and only two more age groups (60–69 and 70–79) could be used for statistical analysis due to the presence of only one patient in other groups (80–89 and 90–99). A continuous decline of the actual number was detected in all of the cell subsets except pericytes among these age groups; male ADSCs 2.4E+3 (50–59); 1.8E+3 (60–69), 1.2E+3 (70–79); male HSC-progenitors 1.6E+3 (50–59); 1.1E+3 (60–69), 700E+0 (70–79); male AT-ECs 368E+0 (50–59); 143E+0 (60–69), 128E+0 (70–79); male pericytes 171E+0 (50–59); 172E+0 (60–69), 51E+0 (70–79). All of the cancer patients were also included in the analysis and no statistically significant difference was observed among 4 age groups as shown in the Fig. 3d. The average BMI for all of the participants was 25.5. The BMI was compared for two groups: BMI ≤ 25 and BMI > 25. Lean persons were defined with BMI < 25 [46]. The average BMI for the group BMI ≤ 25 was 21.3 and for BMI > 25 it was 29.7. Although it was not significant, the average total number for ADSCs and HSC-progenitors were higher in BMI > 25 group when compared to BMI ≤ 25 group. The average numbers for BMI > 25 groups were ADSCs: 4.6E+3, HSC-progenitors: 3.1E+3, AT-ECs: 716.6E+0, and pericytes 145.2E+0. On the other hand, the average numbers for 25 ≤ BMI groups were ADSCs: 3.2E+3, HSC-progenitors: 1.3E+3, AT-ECs: 612.5E+0, and pericytes 127.5E+0 (Data not shown). Over all, Fig. 3 summarizes the correlation of absolute number, with the gender, and age of the donors in the cancer versus non-cancer patients. In conclusion, the donor characteristics of gender, to some extent age, and BMI of the adipose tissue donor, and their physiological condition such as cancer or non-cancer, have the potential to impact the total number of isolated adipose-specific cells. These findings are similar to those obtained by most of the other groups [44, 45].

**Table 2:** The absolute number of isolated cell subsets in 44 non-cancer and 14 cancer patients

Patient number	BMI	Total ADSCs number/cc of fat	Total HSC-progenitors number/cc of fat	Total AT-ECs number/cc of fat	Total pericyte number/cc of fat	DN ratio (%)
1	22.1	354.6E+0	391.0E+0	85.0E+0	8.0E+0	80–98
2	N/A	N/A	N/A	N/A	N/A	5–15
3	14.7	N/A	N/A	N/A	N/A	5–15
4	26.5	3.0E+3	1.5E+3	268.8E+0	107.5E+0	< 2
5	23.1	2.0E+3	924.0E+0	37.6E+0	37.2E+0	80–98
6	20.6	1.5E+3	192.0E+0	391.9E+0	42.4E+0	30–70
7	25.1	1.2E+3	630.0E+0	425.0E+0	23.5E+0	80–98
8	22.4	2.3E+3	1.7E+3	189.0E+0	141.1E+0	< 2
9	27.2	7.6E+3	4.8E+3	2.1E+3	38.4E+0	< 2
10	27.6	3.4E+3	2.1E+3	792.0E+0	61.7E+0	< 2
11	17.2	9.4E+3	2.1E+3	1.7E+3	252.0E+0	< 2
12	23.5	6.1E+3	5.0E+3	2.3E+3	71.9E+0	< 2
13	35.8	1.4E+3	672.0E+0	434.2E+0	40.1E+0	< 2
14	21	2.8E+3	3.1E+3	351.0E+0	328.5E+0	< 2
15	27.8	5.4E+3	5.0E+3	400.8E+0	521.8E+0	< 2
16	24.3	3.9E+3	751.0E+0	260.6E+0	142.1E+0	< 2
17	27.2	568.8E+0	456.9E+0	114.4E+0	19.6E+0	< 2
18	35.9	108.0E+0	172.8E+0	21.6E+0	785.0E+0	80–98
19	25.8	8.6E+3	2.4E+3	63.0E+0	58.2E+0	< 2
20	25.6	8.3E+3	2.5E+3	735.6E+0	40.7E+0	5–15
21	30.1	959.0E+0	1.1E+3	137.5E+0	523.4E+0	5–15
22	39.9	5.1E+3	2.1E+3	1.3E+3	218.4E+0	< 2
23	23.4	8.5E+3	1.7E+3	1.7E+3	381.9E+0	< 2
24	34.4	1.4E+3	826.5E+0	273.1E+0	164.2E+0	< 2
25	24.3	220.2E+0	34.6E+0	3.7E+0	1.6E+0	> 99
26	28.1	88.8E+0	127.2E+0	1.6E+0	25.2E+0	80–98
27	26.6	26.4E+3	28.0E+3	2.5E+3	5.0E+0	< 2
28	23.4	679.2E+0	540.0E+0	46.7E+0	50.1E+0	5–15
29	17.9	524.8E+0	280.0E+0	16.0E+0	163.2E+0	80–98
30	20	1.6E+3	460.0E+0	390.9E+0	16.4E+0	5–15
31	21.6	8.5E+3	3.3E+3	2.2E+3	149.4E+0	80–98
32	20.5	11.6E+3	4.4E+3	1.1E+3	459.4E+0	< 2
33	34.3	10.9E+3	8.4E+3	1.4E+3	96.0E+0	5–15
34	25.1	8.8E+3	1.9E+3	3.8E+3	176.8E+0	> 99
35	32	69.1E+0	64.8E+0	5.0E+0	66.7E+0	> 99
36	23.4	1.9E+3	528.0E+0	1.0E+3	64.7E+0	< 2
37	32.7	15.4E+0	207.9E+0	6.2E+0	3.1E+0	< 2
38	19.8	295.2E+0	28.8E+0	7.2E+0	72.0E+0	> 99
39	24.7	1.9E+3	213.8E+0	427.7E+0	79.2E+0	< 2
40	21	1.5E+3	487.2E+0	311.8E+0	49.7E+0	30–70
41	27.2	1.3E+3	440.2E+0	77.0E+0	51.7E+0	< 2
42	29.7	2.2E+3	1.2E+3	229.7E+0	21.1E+0	80–98
43	22.3	806.3E+0	121.4E+0	25.1E+0	47.9E+0	5–15
44	19	642.2E+0	23.9E+0	281.7E+0	118.3E+0	> 99
45	N/A	1.2E+3	106.0E+0	29.9E+0	9.9E+0	80–98
46	N/A	1.9E+3	3.4E+3	480.5E+0	38.6E+0	5–15
47	N/A	1.6E+3	2.3E+3	166.2E+0	241.8E+0	5–15
48	N/A	1.2E+3	1.1E+3	104.3E+0	80.9E+0	30–70
49	N/A	140.0E+0	224.2E+0	7.8E+0	40.2E+0	< 2
50	N/A	1.5E+3	2.2E+3	168.5E+0	114.1E+0	< 2
51	N/A	3.4E+3	3.1E+3	1.6E+3	71.1E+0	< 2
52	N/A	1.7E+3	2.0E+3	91.1E+0	23.0E+0	< 2
53	N/A	2.1E+3	1.2E+3	169.0E+0	88.1E+0	80–98
54	N/A	500.8E+0	117.0E+0	46.8E+0	88.9E+0	> 99
55	N/A	221.4E+0	87.4E+0	3.4E+0	2.7E+0	< 2
56	N/A	2.2E+3	92.9E+0	108.0E+0	10.8E+0	> 99
57	N/A	729.0E+0	324.0E+0	32.4E+0	25.9E+0	30–70
58	N/A	506.9E+0	244.0E+0	69.7E+0	66.4E+0	80–98

### Culture expansion of SVFs and their differentiation potency

**Fig. 4:**
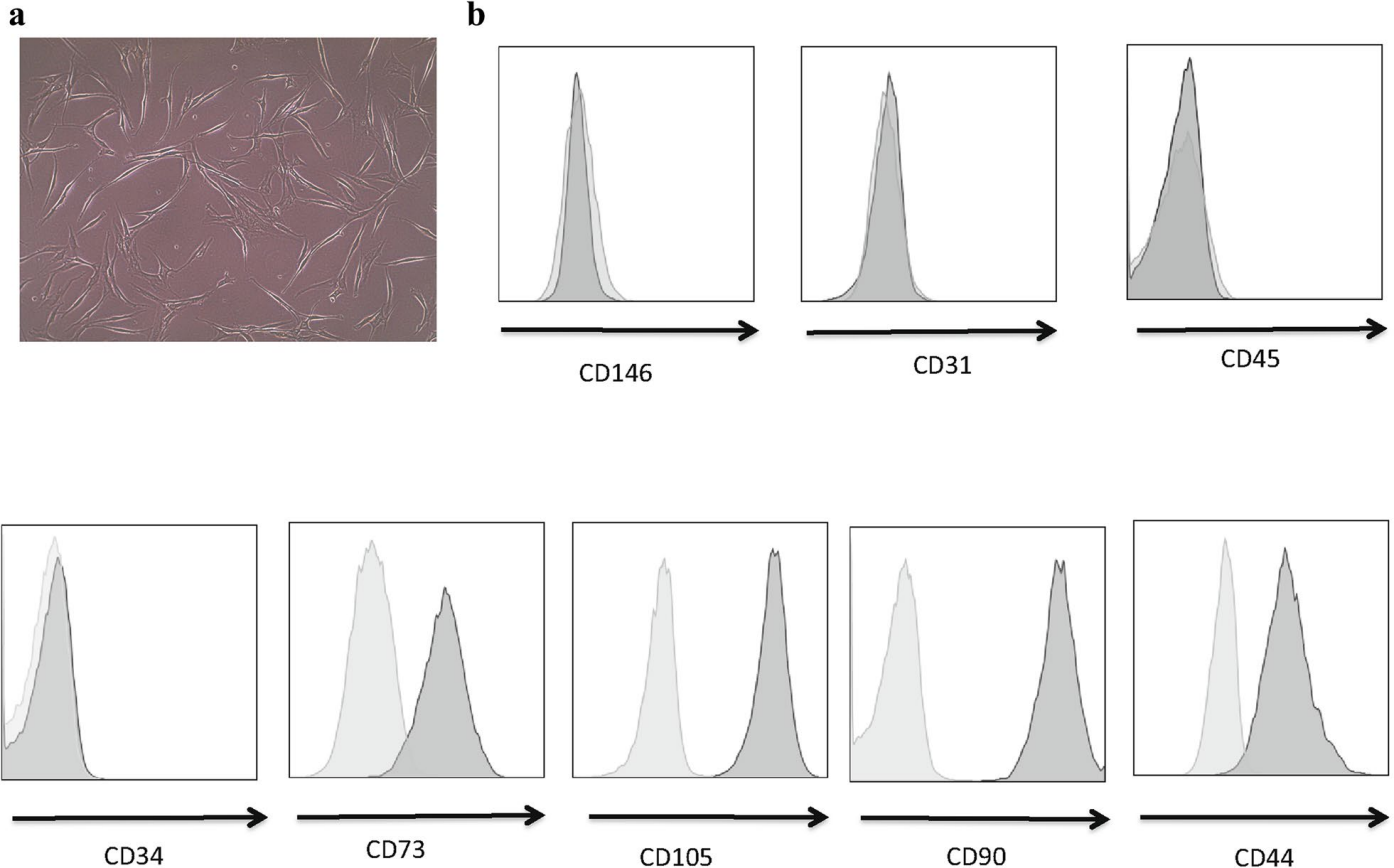
In vitro culture of adipose tissue-derived cells and their flow cytometry analysis. **a** The image of adipose tissue-derived cells in culture showing fibroblast-like morphology following their adherence to the plastic (Passage 0), X10 magnification. **b** Representative histograms show adipose-derived and expanded-mesenchymal stem cells are a homogeneous population of cells and express standard ADE-MSCs specific surface markers, including CD73, CD105, CD90, and CD44, and are negative for CD146, CD31, CD45 and CD34 (dark gray filled). Light gray filled lines represent unstained samples

**Fig. 5:**
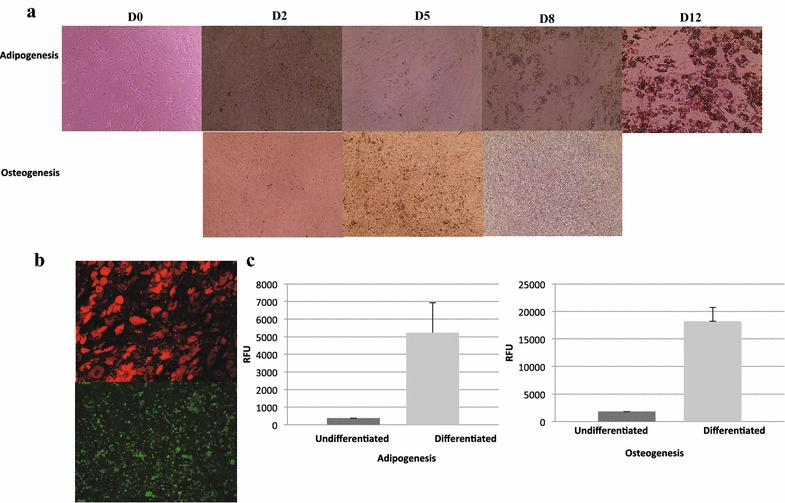
The differentiation potency analysis of ADE-MSCs. **a**, **b** Morphological analysis of in vitro differentiation of ADE-MSCs (P1) under adipogenic and osteogenic media conditions using light microscopy (**a**) and fluorescence microscopy (**b**). **c** The quantification of the accumulation of intracellular triglycerides and calcium deposit in adipogenesis and osteogenesis, respectively. RFU represents relative fluorescence unit read in both of the culture conditions; undifferentiated versus differentiated. Error bars are standard deviations from four independent samples. The P values were calculated using the Student t test. (*p < 0.05 for adipogenesis and **p < 0.02 for osteogenesis)

The majority of the SVF samples were used for treatment in the clinics and a relatively minor fraction was sent to us for further analysis. When there were enough samples, a portion of the cells sent for characterization was also used for cell culture analysis. Cellular expansion of adherent cells was initiated in a culture medium supplied with 5% Stemulate and adipose-derived and expanded-mesenchymal stem cells (ADE-MSCs) cultures were further analyzed for morphology, phenotype, and differentiation. These cells exhibited the expected fibroblast-like morphology following their adherence to the plastic (Fig. 4a) and continuous culture resulted in phenotypically homogeneous population as periodically verified by flow analysis (Fig. 4b). Phenotypic characterization of cultured cells revealed that all of the samples shared the same markers, which have been described, by International Federation of Adipose Therapeutics and Sciences (IFATS) and the International Society for Cell Therapy (ISCT) for the minimum criteria for identifying MSCs. They highly expressed CD90, CD73, CD105, and CD44, while remained negative for CD45, CD146, CD31, and CD34 [22]. There was no variation between the phenotypes of cells from different passages or freezing–thawing cycles. The last criterion to identify ADE-MSCs is to test their multipotency by differentiating into different cell types such as osteoblasts, and adipocytes. Therefore, in order to confirm the mesenchymal stemness of expanded cells, their differentiation potential was tested. ADE-MSCs from different donors at 0–1 passages were induced to differentiate into adipocytes or osteoblasts for 8–14 days in defined culture conditions. The cells started to differentiate into adipocytes as early as day 3 and were fully differentiated at day 14 as consistent with increased cell vacuolation of fatty acid observed under the light microscope using Oil O Red staining (Fig. 5a). The following figure (Fig. 5b) shows the partition of fat droplets of differentiated adipocytes as detected by AdipoRed reagent at day 14. The differentiation was further confirmed by quantification of the accumulation of intracellular triglycerides using the same reagent and measuring the fluorescence in four independent experiments. Relative fluorescence unit (RFU) was 14-fold higher in induced cells (5240 ± 1690) compared to non-induced cells (365 ± 233). Culturing the cells under osteogenic condition induced the cells to differentiate within a week. Osteogenic differentiation (Fig. 5a, b) was confirmed by the formation of aggregates and osteoimage mineralization assay. At day 8, the average calcium deposit in osteoblast reached into tenfold difference compared to undifferentiated control (18,241 ± 2480 versus 1800 ± 430) as quantified by RFU on a plate reader using 492 nm excitation and 520 nm emission wavelengths.

### Expanded MSC and their therapeutic potential

**Fig. 6:**
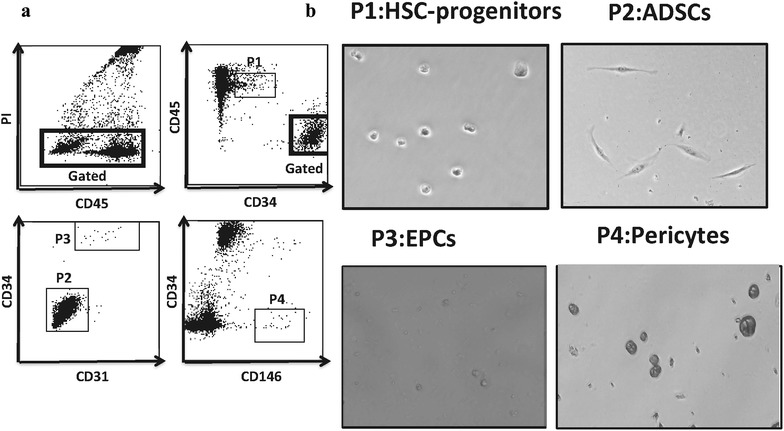
Analyzing the source of culture expanded-mesenchymal stem cells. **a** The four candidate progenitor cell populations were simultaneously sorted and put into a culture according to the shown gating strategy. PI negative live cells were gated. Each region shows sorted population (P) P1; HSC-progenitors, CD34^low^ CD45^+^ CD31^−^ CD146^−^, P2; ADSCs, CD34^high^ CD45^−^ CD31^−^ CD146^−^, P3; AT-ECs, CD34^high^ CD45^−^ CD31^+^CD146^+^, P4; Pericytes, CD45^−^CD34^−^CD31^−^CD146^+^. **b** Representative bright field pictures of sorted SVF populations after 5 days in culture (n = 3)

Understanding the key features of the cellular components of SVF is vital for appreciation of the potential uses and contributions in tissue maintenance and repair. Therefore, we wanted to know which cell subset(s) gave rise to the expanded cell populations. Stem cell (ADSCs and HSC-progenitors) components as well as EC and pericytes of fresh SVF were simultaneously sorted and individually put into culture to observe expansion. Viable (PI-) single cells (height-to-width ratio) were isolated to purity close to 100% (Fig. 6) and put into 48-well plates coated with either Fibronectin or CELLstart CTS. Among 4 different subsets, only CD34^high^ CD45^−^ CD31^−^ CD146^−^ cells formed fibroblast-like morphology and started to divide. This led to the conclusion that ADSCs represent an origin of the ADE-MSC grown in culture, a finding that has been validated by other groups [47–49]. Although neither pericytes nor HSC-progenitors expanded, Trypan blue exclusion analysis determined that 40–60% of the adhered cells were still alive up to 5–7 days post-culture. Next, we asked whether the therapy with expanded cells was as efficient as SVF treatment and evaluated the therapeutic potential of autologous MSC cultured under GMP conditions by American CryoStem. Table 3 summarizes patient’s gender, age, medical condition, the route of injection, injected total number of cells, and patient’s satisfaction assessment. The overall average response rate was 3.1 and did not show a significant difference from the response rate (2.7) observed in SVF treatment group. Therefore we concluded that the therapy with expanded MSC cells originating from CD34^+^ subset was not more efficacious compared to SVF treatment.

**Table 3:** List of 13 ADE-MSCs-treated patient’s gender, age, medical indication, delivery methods and benefit index

Patient number	Gender: F/M	Age	Medical indication	Total number of ADE-MSCs injected (× 10^6^)	Delivery method	Benefit index
59	M	84	Parkinson	40	IV	3
60	F	57	ALS	100	IV	2
61	F	41	MS	10	Ommaya: 4 M IV: 6 M	2
62	M	92	Stroke	80	IV	4
63	F	55	MS	135	Ommaya: 60 M IV:75 M	2
64	M	94	Neuropathy	40	IV	5
65	M	66	Peyronie’s	26	Penile injection	4
66	M	80	Cardiac	10	IV	3
67	F	82	Wellness/lyme disease	30	IV	4
68	M	12	Anoxic brain injury	20	IV	4
69	M	74	Parkinson	30	IV	4
70	M	69	Erectile dysfunction	10	Penile injection	1
71	F	57	ALS	100	IV	2

### The ratio of 4 subsets in adipose tissue derived SVF injected back into the patient

Although the patient’s characteristic we followed seemed to affect the SVF yield, they did not show a correlation with the treatment efficacy. We also checked the SVF cell composition and found out that a particular cell subset did not play a dominant role in the positive clinical outcome. In our cell culture experiments, only the ADSC component of SVF could give rise to expanded cell populations. Furthermore, the therapy with expanded MSCs did not suggest more efficacies compared to SVF treatment. The question remains; do other cell subsets directly contribute to tissue repair/regeneration as a result of a complex interactive network? Since all of the patients were given a combination of cell subsets, we asked whether a certain combination rather than individual subset might play a role in determining the treatment efficacy. We first explored the relative ratios of total injected numbers of ADSC: HSC-progenitors: AT-ECs: Pericytes. Since the number of pericytes made the lowest portion among these 4 subsets, we started our analysis by calculating the ratio based on pericytes. However, we couldn’t derive any meaningful conclusion (data not shown). Therefore, the pericytes were excluded and the relative ratios were next calculated based on 3 subsets. Table 4 shows the ratio of ADSCs: HSC-progenitors: AT-ECs, based on total number of each injected cell subset.

**Table 4:** The ratio of ADSCs, HSC-progenitors, and AT-ECs based on total number of each injected cell subset

Patient number	Delivery method: Direct (localized) injection (DI) or I.V	Benefit index	Ratio ADSCs: HSC-progenitors: AT-ECs (based on total number of each injected fraction)	The Ratio of ADSCs to HSC-progenitors
3	N/A	5	N/A	N/A
1	IV + DI	4	4:5:1	0.8
8	IV + DI	4	12:10:1	1.2
9	IV + DI	4	4:3:1	1.33
10	IV + DI	4	4:3:1	1.33
15	IV + DI	4	14:13:1	1.08
17	IV + DI	4	5:4:1	1.25
18	IV + DI	4	5:8:1	0.63
22	IV	4	4:2:1	2.00
25	IV	4	60:9:1	6.67
32	IV	4	11:4:1	2.75
33	IV + DI	4	7:6:1	1.17
Mean ratio of ADSCs to HSC-progenitors				*1.84*
6	DI	3	4:0.5:1	8.00
12	IV + DI	3	3:2:1	1.50
13	IV + DI	3	3:2:1	1.50
14	IV + DI	3	8:9:1	0.89
16	DI	3	15:3:1	5.00
24	IV	3	5:3:1	1.67
35	IV + DI	3	14:13:1	1.08
36	IV + DI	3	2:0.5:1	4.00
41	IV + DI	3	17:6:1	2.83
42	IV + DI	3	10:5:1	2.00
Mean ratio of ADSCs to HSC-progenitors				*2.85*
2	N/A	2	N/A	N/A
4	IV	2	11:6:1	1.83
20	IV + DI	2	23:3:1	7.67
27	IV + DI	2	11:11:1	1.00
44	IV + DI	2	2:0.1:1	20.00
Mean ratio of ADSCs to HSC-progenitors				*7.63*
21	IV	1	3:1:1	3.00
23	IV	1	5:1:1	5.00
26	IV + DI	1	56:80:1	0.70
28	IV	1	15:12:1	1.25
30	IV + DI	1	4:1:1	4.00
31	IV + DI	1	4:1:1	4.00
34	IV + DI	1	2.5:0.5:1	5.00
40	DI	1	5:1.5:1	3
43	IV + DI	1	33:5:1	6.7
Mean ratio of ADSCs to HSC-progenitors				*3.63*
5	IV	N/A	55:25:1	2.20
7	IV	N/A	3:2:1	1.50
11	IV + DI	N/A	6:1:1	6.00
19	IV + DI	N/A	272:38:1	7.16
29	IV + DI	N/A	33:18:1	1.83
37	IV + DI	N/A	3:34:1	1.0
38	IV	N/A	41:4:1	10.25
39	IV + DI	N/A	5:0.5:1	10.00
Mean ratio of ADSCs to HSC-progenitors				*4.99*

**Fig. 7:**
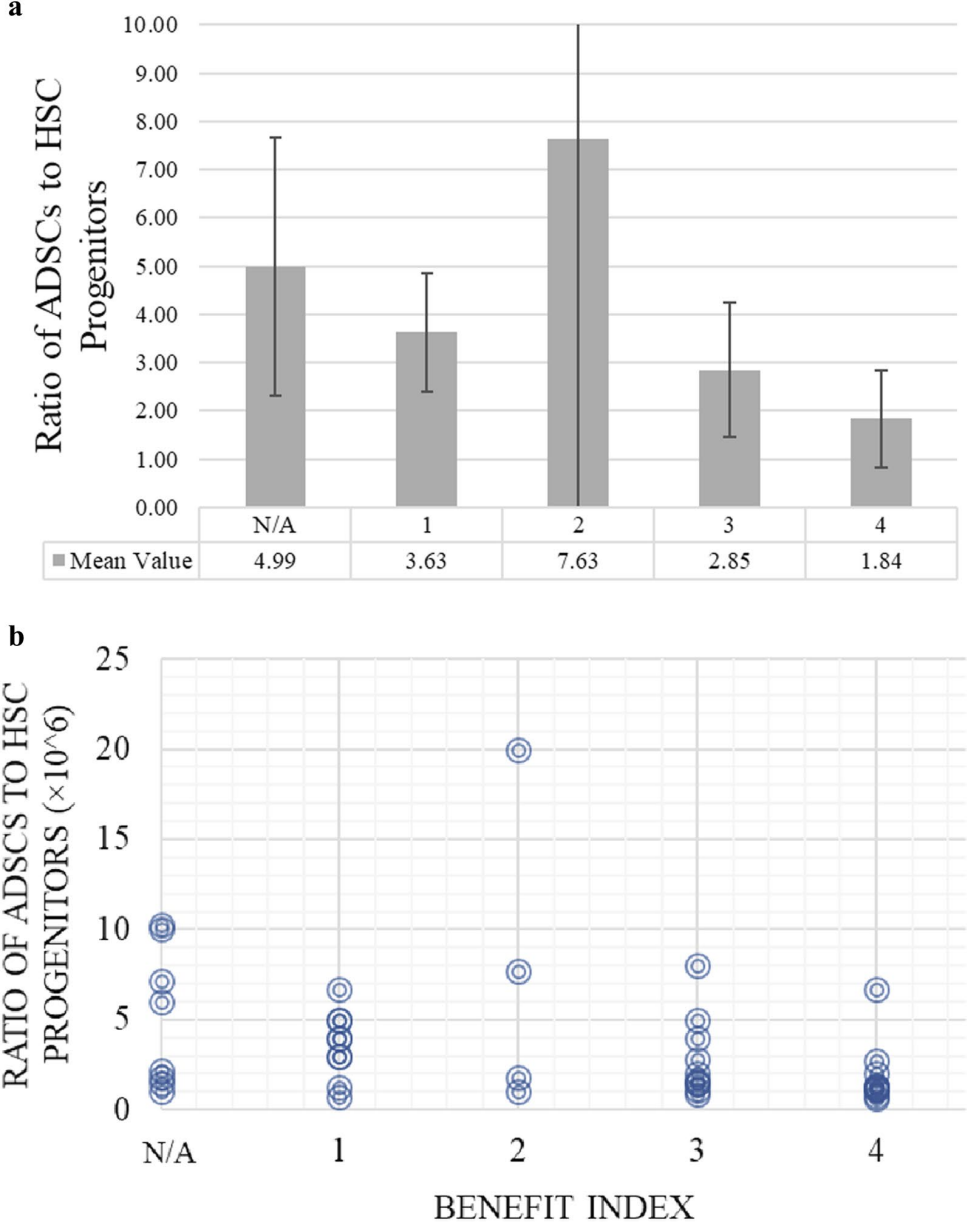
Analyzing the ADSC: HSC-progenitors ratio and correlating with the benefit index. **a** The mean ratio of ADSC’s to HSC progenitors is shown as the height of the bars, and the benefit index is shown as the X-axis. The error bars represent the 95% confidence interval. **b** The raw data from which **a** was derived is shown as a scatter-plot, with the ratio as the Y-axis and the reported benefit index as the X-axis. The Y-values are shown as × 10^6^, and a downward trend is clearly visible in **a**, suggesting a correlation between the ratio and the benefit of the treatment

The degree of the ratio of ADSCs to HSC-progenitors in the given treatment was associated with overall treatment efficacy, whereas their lower ratio resulted in a higher benefit index on average. The data from Table 4 are summarized in Fig. 7. A downward trend can be seen between the benefit index and the mean ratio as the benefit index increases (Fig. 7a). The error bars represent the 95% confidence interval. The total average of the ratio of ADSCs to HSC-progenitors in patients who benefited highly from the treatment was 1.84, which signifies that every HSC-progenitor should be accompanied by at least two ADSCs to achieve the maximum patient benefit. For the next groups of patients with the benefit indexes of 3 and 2, the average for the ratio became 2:85 and 7.73, respectively. The patients who reported no benefit, and the patients who did not report back to the clinic, both shared a close average ratio value of approximately 4 (3.63 and 4.99, respectively). In another separate representation the raw data from which (A) was derived was plotted for every single patient (Fig. 7b). Downward trend is again clearly visible suggesting a correlation between the ratio and the benefit of the treatment.

## Discussion

There is already an abundant number of clinical trials and published research on the uses of adipose derived stem cells on a wide range of diseases and conditions. Data suggest that stem cell treatment either in the non-cultured, unexpanded form as SVF or in the culture-expanded form as MSC is safe. However, there is a paucity of evidence explaining clinical efficacy variations and explaining the mechanism of action of transplanted cells. There is a speculation that the potency of autologous stem cell treatment may vary due to cell isolation methods, heterogeneity of the patients reflected as age, disease type and state. However, the data are insufficient to establish a reliable dose and mode of administration versus effect relationship. Therefore, in this study we mainly focused on determining the factors that influence true effectiveness of the treatment in order to delineate the most clinically effective usage of SVF.

Stromal vascular fraction is an attractive treatment method because of the easy isolation of autologous SVF from 50 cm^3^ of adipose tissue lipoaspirate in a sterile closed system [14]. The harvesting procedure takes 1–2 h allowing the injection to take place in a very short period of time without any alterations. After digestion of the tissue and removal of differentiated adipocytes, the so-called SVF, a mix of various cell types, is obtained. However, it is known that SVF contains a small percentage of ADSCs, estimated at 1–10% of the SVF [43], data that are also confirmed by us when blood contaminants, and all other stromal cells present in SVF were taken into the consideration. However, our data strongly suggest that not only blood contaminants (CD45^+^CD34^−^), but also DNs (fibroblasts, pre-adipocytes, smooth muscle, mature endothelial cells, the remaining RBCs) are likely the cause of higher variation among the SVF samples. Therefore we believe that the presentation of the adipose-resident cell subsets as percentage could be misleading. Parting from other studies, we followed a different methodological approach and focused on identification and enumeration of SVF subpopulations. Flow analysis showed that freshly isolated SVF was very heterogeneous and harbored different stem cell and precursor/progenitor components as well as mature epithelial cells and PBMCs [20, 21]. As previously described by Zimmerlin et al., and others, we identified four major stem cell and precursor/progenitor subsets [19, 39]. They were specific to adipose tissue since they were not detected in the blood of the patients. Based on post-isolation absolute number CD34^high^ CD45^−^ CD31^−^ CD146^−^ ADSCs was the major subset, followed by CD34^low^ CD45^+^ CD206^+^ CD31^−^ CD146^−^ HSC-progenitors. ADSCs were documented as conventional multipotent adventitial cells while HSC-progenitors were described to be superior to ADSCs to induce angiogenesis and be differentiated into vascular endothelial cells [19, 41]. CD34^high^ CD45^−^ CD31^+^CD146^+^ AT-ECs and CD45^−^CD34^−^CD31^−^CD146^+^ pericytes made the third and forth subsets, which were detected in lower numbers. Pericytes, which were initially thought to be a progeny for the less primitive ADSCs, compromised less than 1% of SVF. In a couple of studies, hematopoietic progenitor cells and pericytes, were shown to possess characteristics similar to ADSCs, and therefore could grow in adherent culture and displayed mesenchymal multipotency [25, 50, 51]. A recent study by Hardy et al. created a heatmap displaying hierarchical clustering among genes representative of ADSCs and pericytes and suggested that a higher percentage of ADSCs exhibit a stem-like phenotype as compared to pericytes [52]. Following the sorting and culturing of 4 individual subsets, the only cell subset, which gave rise to adipose-derived and expanded-mesenchymal stem cells in our study, was originated from the conventional ADSCs subset. It should be noted that this definition of ADE-MSCs applies to plastic adherent cultured and not freshly isolated ADSCs. There are a couple of different explanations as to why the other subsets did not expand. First of all, the majority of the SVF samples were used for treatment and a relatively minor fraction was sent to us for further analysis. Therefore, we were very limited in sample size in order to get enough number of cells after sorting and/or optimize the culture conditions for the growth of any particular subset. Because of this limitation, the culture media condition (DMEM + %5 Stemulate) was kept consistent for all sorted cells in order to identify the source of culture-expanded MSCs under such condition. Furthermore SVF samples used for sorting has always provided the lowest amount of CD34^+^CD31^+^AT-ECs and CD146^+^ pericytes which might be quiescent or may not reach enough number to induce an expansion partly because of limited cell number and/or the differential SVF processing compared to other methods. Secondly, adipose derived cells have been traditionally expanded with FBS as part of the culture media. However, in our study we used 5% Stemulate (clinically compliant pooled human platelet lysate) to provide growth factors and other proteins to support proliferation of the sorted cells. Any change in tissue culture practices is likely to contribute to the differential growth of lineage cells of different origin.

Previous studies suggested that factors such as age, gender, BMI, lesion or defect size, or stage of the medical condition could be important in modulating the benefit of stem cell therapies [42–45]. We, on the other hand, initially hypothesized that the abundance of one or more of these subsets were the primary factors, which could determine the treatment outcome. ADSC counts varied widely between our patients, and the enumeration analyses suggest that the hypothesis that patient’s factors such as gender, to some extent age, and even physiological condition such as cancer versus non-cancer of the adipose tissue donor may have the potential to impact the total number of isolated adipose-specific cells. However, the same criteria did not play a role on the clinical outcome using our current technique and approach. Since we used the same standard optimized technique to process the fat for each patient, the functionality and quality of the derived cells were expected to maintain among all of our patients. Regarding what was observed and measured as a significant improvement including the methods of comparison there is variability in the literature [reviewed in 53]. However, symptom relief, especially the pain reduction and increased mobility and function were frequently used as the main criteria to report a successful outcome [8, 54]. For the assessment, we used the same evaluation forms, questionnaires, and score index, tests in addition to the patient’s additional descriptive information on pain, quality of life, and physical functioning. However, we cannot rule out the fact that there could be some patient bias to some degree, when they fill out the survey forms for the follow-up.

## Conclusions

Autologous cell therapy holds promise for a nearly unlimited variety of different chronic diseases and degenerative or traumatic conditions. The concept of “drug” therapy is the use of one chemical agent that is intended for a generally single purpose to optimize physiologic conditions resulting in the mitigation of cellular damage in an effort to promote healing. However the concept of “cell therapy” significantly differs since live stem cells could potentially be directly responsible for healing either through direct engraftment or cellular communication. It has been acknowledged that SVF has a promising capacity as a tool for regenerative medicine and recently, a new debate on the regenerative effects of individual SVF cell subsets has been introduced [1, 6, 55]. It is still unclear how each cell subset either individual or together, shows their effect at the site. It is possible that ADSCs can differentiate into mature cells and/or mediate a therapeutic benefit through cytokine, chemokine, paracrine-driven mechanisms inducing angiogenesis, cell proliferation and anti-inflammatory responses thorough a network of other SVF cells. Moreover, in addition to soluble factors, MSCs also secrete membrane-derived extracellular microvesicles (ExMVs), which may deliver messenger RNA, micro RNA, and proteins in order to communicate with the surrounding cells and orchestrate several biological processes [56]. Our data support the notion that a particular SVF-dose, with a certain composition/combination, may increase the clinical outcome benefit. The interaction of ADSCs and HSC-progenitors along with AT-ECs in a specific ratio could lead to the conditioning of each other for more efficient secretion of soluble factors, thereby increasing the healing potential of the injected cells. We believe that EC is an important contributing factor since we took the ADSC/HSC ratio per unit of EPC in our calculations and statistical analysis. Consequently, we think EC’s role is very vital in terms of providing blood supply or vascular network. If these autologous healing cells can be made more bio-available by isolating them and re-introducing them in large numbers into or near damaged tissue, then such deployments are clinically justified for a very wide variety of disease conditions. Fresh autologous stromal vascular fraction containing a rich mixture of various regenerative cell lines can be easily isolated in the operating room and redeployed back into patients during the same procedure to achieve the desired increase in stem cell bioavailability. These findings open the way of tailored design of new treatment regimen for individuals by adjusting the cells ratio before the treatment. However, preparation of standardized concentrate for SVF injections could be cumbersome and limited mainly because of FDA regulations and other concerns in handling. To our knowledge, this is the first report showing that the stem cell’s composition may have a predictive value for the treatment response. These findings permit further investigations to both better characterize this association in larger cohorts and begin to elucidate the underlying mechanism(s) of this phenomenon.

## Abbreviations

SVF: stromal vascular fraction
ADSCs: adipose-derived stromal/stem cells
HSC-progenitors: hematopoietic stem cell-progenitors
AT-ECs: adipose tissue-endothelial cells
EPCs: endothelial progenitor cells
FACS: fluorescence-activated cell sorting
MSCs: mesenchymal stem cells
ADE-MSCs: adipose-derived and expanded-mesenchymal stem cells
HSC: hematopoietic stem cells
GMP: good manufacturing practices
cc: cubic centimeter
7-AAD: 7-amino-actinomycin D
PI: propidium Iodide
BMI: body mass index
D.I: direct localized injection
I.V: intravenous
DN: double negatives
IFATS: International Federation of Adipose Therapeutics and Sciences
ISCT: International Society for Cell Therapy
RBC: red blood cells
GMP: good manufacturing practice
RFU: relative fluorescence unit
ExMVs: membrane-derived extracellular microvesicles
FDA: Food and Drug Administration
FBS: fetal bovine serum
PDGF: platelet-derived growth factor
EGF: epidermal growth factor
VEGF: vascular endothelial growth factor
bFGF: basic fibroblast growth factor
TGF-B: transforming growth factor beta
RANTES: regulated upon activation, normal T cell expressed, and secreted
CCL: chemokine ligand
CXCL: C-X-C Motif Chemokine Ligand
